# Systematic review and meta-analysis of microbiota-gut-astrocyte axis perturbation in neurodegeneration, brain injury, and mood disorders

**DOI:** 10.1016/j.bbih.2025.101013

**Published:** 2025-05-12

**Authors:** Daniel E. Radford-Smith, Katharine Oke, Carolina F.F.A. Costa, Daniel C. Anthony

**Affiliations:** Department of Pharmacology, University of Oxford, Mansfield Road, Oxford, OX13QT, UK

**Keywords:** Astrocytes, GFAP, Depression, Alzheimer's disease, Parkinson's disease, Stroke, Traumatic brain injury, Microbiota-gut-brain axis

## Abstract

**Background:**

Astrocytes are essential for preserving homeostasis, maintaining the blood-brain barrier, and they are a key element of the tripartite neuronal synapse. Despite such multifaceted roles, their importance as contributors to the microbiota-gut-brain axis studies, which typically focus on microglia and neurons, has been largely overlooked. This meta-analysis provides the first systematic review of the microbiota-gut-astrocyte (MGA) axis *in vivo,* integrating findings across distinct neurological diseases.

**Methods:**

A systematic narrative review was conducted per PRISMA guidelines. The search term employed for PubMed was *“Microbiota"[MeSH] AND (astrocyte OR glial) NOT (Review[Publication Type])* and for Web of Science, Embase, and Scopus, *“Microbio∗ AND (astrocyte OR glial)”* with filters applied to exclude review articles. Searches were completed by May 9th^,^ 2024. Data extracted included study models, interventions, and outcomes related to astrocyte biology and rodent behaviour. SYRCLE's risk of bias tool was used to assess individual study designs.

**Results:**

53 studies met the inclusion criteria, covering rodent models of stroke and traumatic (acute) brain injury, chronic neurodegenerative diseases including Alzheimer's and Parkinson's disease and other heterogeneous models of cognitive impairment and affective disorders. Significant heterogeneity in methodology was observed between studies. Five studies had a high risk of bias, and 15 were low risk. Astrocyte biology, typically measured by GFAP expression, was increased in neurodegeneration and acute brain injury models but varied significantly in mood disorder models, depending on the source of stress. Common findings across diseases included altered gut microbiota, particularly an increased Bacteroidetes/Firmicutes ratio and compromised gut barrier integrity, linked to increased GFAP expression. Faecal microbiota transplants and microbial metabolite analyses suggested a direct impact of the gut microbiota on astrocyte biology and markers of neuroinflammation.

**Conclusions:**

This review and meta-analysis describes the impact of the gut microbiota on astrocyte biology, and argues that the MGA axis is a promising therapeutic target for neurological disorders. However, it is clear that our understanding of the relationship between the gut microbiota and astrocyte behaviour is incomplete, including how different subtypes of astrocytes may be affected. Future studies must adopt new, multi-dimensional studies of astrocyte function and dysfunction, to elucidate their role in disease and explore the therapeutic potential of gut microbiota modulation.

## Highlights

• Changes to astrocyte biology, shown by GFAP expression, link to brain injury, neurodegeneration, and mood disorders.
• Meta-analysis shows stress type alters GFAP: Physiological stress raises it, psychological stress lowers it.
• Gut Dysbiosis across diseases shows reduced barrier integrity and higher Bacteroidetes/Firmicutes ratio.
• Microbiota therapies like probiotics and faecal transplants may restore BBB and reduce neuroinflammation.
• We identify **a se**x and age bias and call for more female and older animals to study MGA axis interactions.

## Graphical abstract

**Figure:**
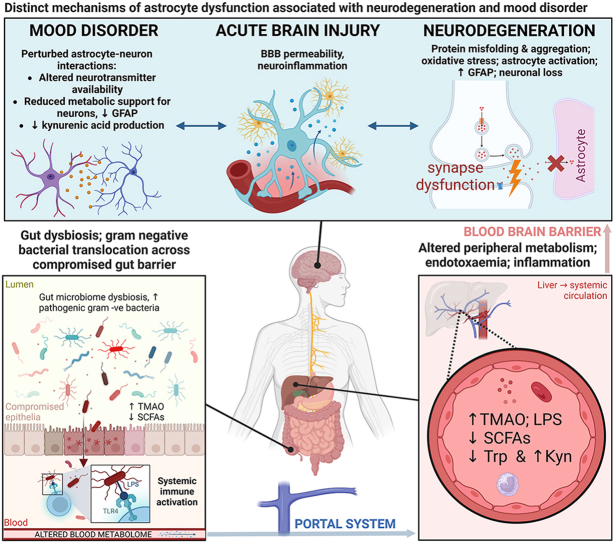

## 1 Introduction

Astrocytes are dynamic, highly interactive cells of neuroectodermal origin and are derived from radial glia *in vivo* (Sofroniew, 2020). As the most abundant brain cell type in mammals (Herculano-Houzel, 2014), astrocytes actively participate in multiple aspects of neurophysiology, including the maintenance of electrolyte balance, neurotransmitter levels, and energy homeostasis to support and maintain brain function (Verkhratsky and Nedergaard, 2018). Anatomically, astrocytes exhibit a complex, highly branched arborisation. Functioning as a reticular syncytium, they form a connected and highly organised network throughout the entire central nervous system (CNS) (Kiyoshi and Zhou, 2019), forming intimate physical and chemical interactions with neuronal synapses (so-called the tripartite synapse) as well as the endothelial cells and tight junctions that maintain the blood-brain barrier (BBB) (Abbott et al., 2006). However, it is becoming clear that astrocyte responses are not restricted to changes within the CNS; their behaviour is also influenced by distal signals, including from the gut microbiota (Sanmarco et al., 2021).

While distinct from “host” cells, microbes have co-existed with mammals throughout evolution, and the gut microbiota, the most dense microbial niche in humans and rodents, exerts considerable influence on host CNS physiology in both healthy and diseased states (Cryan et al., 2019; Diener et al., 2022; Chen et al., 2022). Despite their indispensable role in mediating synaptic transmission and intimacy with the BBB, astrocytes are often overlooked in studies interrogating microbiota-gut-brain interaction. The focus has instead been on interactions between microbes, microglia (resident brain innate immune cells), and neurons (Cook and Prinz, 2022). This systematic review focuses on the microbiota-gut-astrocyte (MGA) axis to emphasise the important yet understudied interactions between gut microbes and astrocytes (Loh et al., 2024).

In response to injury or inflammatory insult to the brain, astrocytes, both fibrous (white matter) and protoplasmic (grey matter), become reactive and upregulate the cytoskeletal component glial fibrillary acidic protein (GFAP). As the prototypical marker for astrocytic response and contribution to neuroinflammation in rodents (Zhou et al., 2019a), it is worth noting that GFAP only reveals the cytoskeletal structure rather than the whole volume of the cell (which is more complex), and can therefore generate misleading information (Zhou et al., 2019a). This molecular phenotype directly relates to the underlying structural changes in astrocyte morphology that parallels astrocyte dysfunction. Specifically, a loss of finer GFAP + processes and, by extension, engagement of synaptic territory by astrocytes precludes the efficient clearance of glutamate and ions from the synaptic cleft, potentiating neuronal excitotoxicity and exacerbating synaptic dysfunction. This behaviour is likely to contribute to the neurodegeneration observed in all pathologies where astrocyte activation is a feature – from Alzheimer's disease (AD) and Parkinson's disease (PD), to multiple sclerosis as well as in the natural ageing process (Zhou et al., 2019a; Patani et al., 2023).

The mechanistic overlap is less clear in other brain disorders such as acute brain injury, or mood disorders where the morphological changes in astrocytes are also much less well-studied. In depression, for example, astrocytes may be “dyshomeostatic” rather than purely reactive (González-Arias et al., 2023). Astrocyte reactivity could be considered a form of “dyshomeostasis”, the important point being that future research must address the complexity of astrocyte function and dysfunction in disease, incorporating multiple dimensions and higher resolutions of astrocyte function (e.g. energy homeostasis informed by an integrated metabolomics and transcriptomics approach). In doing so, rescuing astrocyte morphology and function (astrocyte-synapse connections), potentially through modification of the gut microbiota, becomes an incredibly exciting therapeutic target.

While dysbiosis of the gut microbiome is a common feature across acute brain injury (Cryan et al., 2020), neurodegenerative disease (Cryan et al., 2020), and mood disorder (Amin et al., 2023), connections between the gut microbiome, systemic and brain inflammation, and astrocyte (dys)function remains unclear. This systematic review aims to analyse current studies to address the following key questions:

1. The role of astrocytes: How is astrocyte activity assayed in the literature, and how does it change across different neurological disorders?
a. A meta-analysis of GFAP expression was performed to investigate the changes in astrocyte biology across neurological disorders.
2. Evidence for a causal relationship: Does gut microbiota composition affect astrocyte function?
3. Mechanisms: What are the key factors regulating the MGA axis to affect neuropathology and behaviour?

## 2 Methods

This systematic review and meta-analysis was conducted in accordance with PRISMA guidelines (Page et al., 2021).

### 2.1 Search strategy

PubMed, Web of Science, Embase, and Scopus were screened for relevant *in vivo* studies in rodent models (date of last screening May 08, 2024). The search term employed for PubMed was *“Microbiota"[MeSH] AND (astrocyte OR glial) NOT (Review[Publication Type])* and for Web of Science, Embase, and Scopus, *“Microbio∗ AND (astrocyte OR glial)”* with filters applied to exclude review articles. To capture as many relevant studies as possible, no search terms were included pertaining to specific mouse models and were instead screened for eligibility according to model phenotype at the full-text stage.

### 2.2 Selection criteria

**Systematic review:** The exclusion criteria were review articles, which were excluded at the point of searching. Conference, abstracts, and meeting minutes were excluded at the point of study screening, as were studies that did not involve the gut microbiota or a measure of astrocyte activity/activation. Non-English articles were also excluded. The inclusion criteria were pre-clinical original studies in rodents that investigated a link between the gut microbiome and CNS astrocyte biology and reported behavioural outcomes in a neurodegenerative, neuropsychiatric, or acute brain injury model. Studies were then further grouped according to the phenotype being modelled *in vivo,* including neurodegenerative disorders (AD, PD), mood disorders (depressive, anxiety-like), and acute neuroinflammatory conditions (stroke and traumatic brain injury [TBI]).

**Meta-analysis of GFAP expression:** 44 of the 53 studies included in the systematic review measured GFAP expression and included a control group alongside a relevant disease model and were thus eligible for meta-analysis (Table S2). Due to the heterogeneity regarding other treatments, the meta-analysis was limited to disease vs. control and other treatment arms were not included.

### 2.3 Selection of studies

Titles and abstracts of the articles retrieved via the search strategy were screened by two independent reviewers. Initial screening was followed by a full-text review to assess eligibility according to the full selection criteria. Disputes were resolved by discussion based on the inclusion and exclusion criteria, and if necessary, consultation with a third independent reviewer.

### 2.4 Study quality assessment

The SYRCLE's risk of bias tool for animal studies was used to assess the study design of each included article (Hooijmans et al., 2014). Studies were each given a score out of 10 by a single reviewer and cross-checked by a second independent reviewer. A higher score indicates a lower risk of bias. Discrepancies in scoring were resolved through discussion, and if needed, a third reviewer was consulted (Fig. S1 and Table S1).

### 2.5 Data extraction and statistical analysis

Following the full-text screening process, data extracted from studies that met the selection criteria included authors, publication year, animal (disease) model, study interventions (e.g. diet, inflammatory stimuli), and key outcome measures focusing on behavioural outcomes and effects on astrocyte biology.

Due to the significant degree of heterogeneity between study methodology, including choice of animal model, behavioural analysis, and reporting of microbiota data, a systematic narrative review was used. Both statistically significant and non-significant results have been reported and distinguished in this systematic review.

Data for the meta-analysis of GFAP expression were digitally extracted (automeris.io/wpd). GFAP expression levels were standardised to fold-change relative to the control group. Specifically, means and standard deviations (computed from standard error where necessary) were transformed relative to control values, allowing for consistent comparisons across studies.

Meta-analyses were performed using the meta (Balduzzi et al., 2019) and metafor (Viechtbauer, 2010) packages in R. The effect size for each study was calculated as the standardised mean difference (SMD) between disease and control groups. Random effects models were employed due to expected heterogeneity between studies. Subgroup analyses were conducted for neurodegenerative diseases (Alzheimer's Disease, AD, Parkinson's Disease, PD, and other cognitive decline, CD) and mood disorders (psychological and physiological stressors). The primary outcomes reported include pooled effect sizes with 95 % confidence intervals (CIs), heterogeneity statistics (I^2^), and publication bias assessments using funnel plots and Egger's regression test. Results were visualized using forest plots highlighting pooled effects and subgroup analyses.

## 3 Results

848 unique articles were returned through the search of electronic databases and subjected to title and abstract screening (Fig. 1). Of these articles, 116 full texts were reviewed and 53 studies fulfilled all exclusion and inclusion criteria and were included in this qualitative review (Table S3). Egger's test indicated the potential for publication bias (p = 0.046) for studies included in the meta-analysis of GFAP expression (Fig. S2). Five studies had a high risk of bias, and 15 were low risk. The most common causes of bias across all studies were failure to report measures used (if any) to house animals randomly within the animal room, and whether or not animals were selected at random for outcome assessments.

**Fig. 1:**
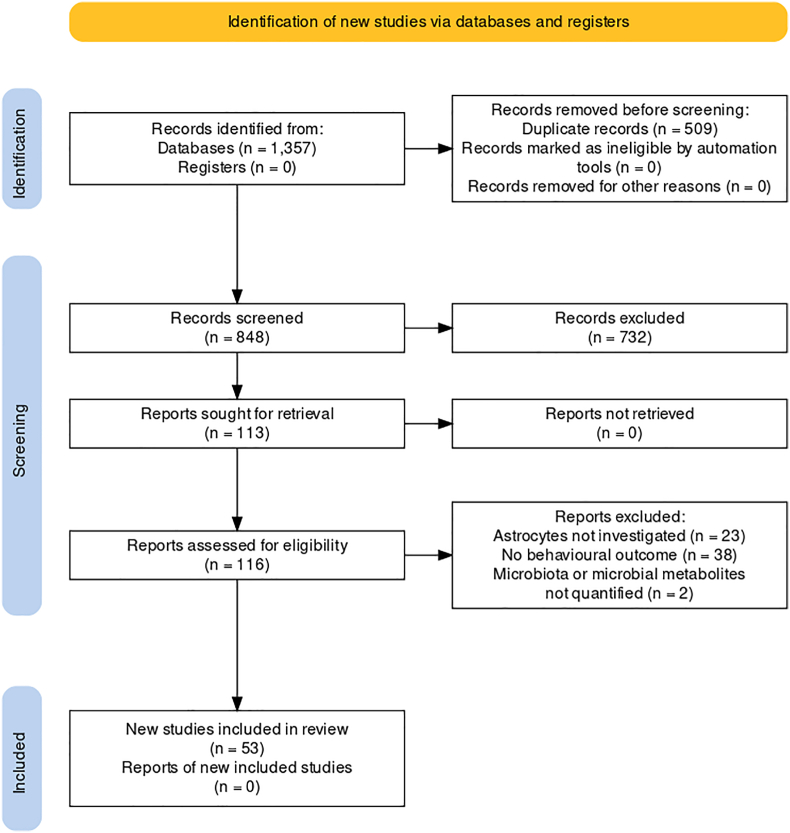
PRISMA flowchart of the literature search and study selection process.

### 3.1 Overview of animal models

The 53 eligible studies broadly fit into rodent models of acute brain injury (stroke (Duan et al., 2024; Li et al., 2022; Zhang et al., 2024a; Silva de Carvalho et al., 2022; Ahnstedt et al., 2020) and acute TBI (Ma et al., 2019)), neurodegenerative disorders and cognitive impairment (AD (Liu et al., 2020; Syeda et al., 2018; Cuervo-Zanatta et al., 2023; Wang et al., 2024; Pan et al., 2022; Wang et al., 2019; Colombo et al., 2021; Qian et al., 2023; Qu et al., 2022; Giridharan et al., 2023; Kim et al., 2020), PD (Jang et al., 2020; Cui et al., 2022; Liu et al., 2022; Wang et al., 2022; Zhou et al., 2019b, 2021, 2024; Fang et al., 2019; Sun et al., 2018; Guo et al., 2023; Dwyer et al., 2021), and other models of cognitive impairment (Hao et al., 2023; Giridharan et al., 2022; Syeda et al., 2022; Gao et al., 2020; Zheng et al., 2023; Wei et al., 2020; Wu et al., 2021; Hoyles et al., 2021; Angoa-Pérez et al., 2020; Shi et al., 2020; Brunt et al., 2021; Tian et al., 2024)), and mood disorders (Li et al., 2021; Xu et al., 2024; Wang et al., 2023a, 2023b; Zhang et al., 2019; Lakosa et al., 2023; Zhou et al., 2023; Radford-Smith et al., 2022; Lv et al., 2019; Huang et al., 2023; Chu et al., 2019; Hosseinifard et al., 2019; Diviccaro et al., 2019).

Four of the five studies investigating stroke outcomes utilised transient middle cerebral artery occlusion (MCAO) for either 30 (Silva de Carvalho et al., 2022), 60 (Ahnstedt et al., 2020, Duan et al., 2024), or 120 (Zhang et al., 2024a) minutes, and one study utilised exogenous intracerebral collagenase to induce acute intracerebral haemorrhage (Li et al., 2022). Post-stroke study endpoints were highly variable between studies including 1 (Duan et al., 2024), 3 (Li et al., 2022), 7 (Zhang et al., 2024a), 42 (Ahnstedt et al., 2020), and 56 (Silva de Carvalho et al., 2022) days. Four of the studies were in mice (Duan et al., 2024; Li et al., 2022; Silva de Carvalho et al., 2022; Ahnstedt et al., 2020) and one study used rats (Zhang et al., 2024a). In the acute TBI model, the weight-drop method was used in mice and brain and behaviour were analysed 3 and 7 days post-injury (Ma et al., 2019). Two studies in stroke did not include a sham group (without MCAO/ICH) when assessing GFAP expression (Li et al., 2022; Silva de Carvalho et al., 2022).

Of the 11 studies that investigated the gut microbiome and astrocyte biology in the context of AD, six studies (Liu et al., 2020; Cuervo-Zanatta et al., 2023; Wang et al., 2019; Colombo et al., 2021; Qian et al., 2023; Giridharan et al., 2023) utilised APP/PS1 transgenic models in mice, one study (Kim et al., 2020) in a relatively new ADLP^APT^ transgenic mouse model (Kim et al., 2018), whilst others utilised a triple transgenic (APPSWE, PS1M146V, TauP301L) mouse model (Syeda et al., 2018), TgCRND8 mouse model (Qu et al., 2022), 5XFAD mouse model (Pan et al., 2022), or a spontaneous (non-genetic) model through bilateral intracerebroventricular beta-amyloid injection in rats (Wang et al., 2024). One study had no WT (non-AD) control group (Giridharan et al., 2023). Of the 11 studies in PD, 10 utilised repeat intraperitoneal injection of the toxin 1-methyl-4-phenyl-1,2,3,6-tetrahydropyridine (MPTP) (Jang et al., 2020; Cui et al., 2022; Liu et al., 2022; Wang et al., 2022; Zhou et al., 2019b, 2021, 2024; Fang et al., 2019; Sun et al., 2018; Guo et al., 2023) with a dose of 20mg/kg or 30mg/kg most commonly daily for 5 or 7 consecutive days, though one study injected 4 times in just one day (Zhou et al., 2019b). Another study utilised intracranial lipopolysaccharide (LPS) injection followed by intraperitoneal paraquat injection (Dwyer et al., 2021). All PD models were in mice.

A further 12 studies (11 in mice (Hao et al., 2023; Syeda et al., 2022; Gao et al., 2020; Zheng et al., 2023; Wei et al., 2020; Wu et al., 2021; Hoyles et al., 2021; Angoa-Pérez et al., 2020; Shi et al., 2020; Brunt et al., 2021; Tian et al., 2024), one in rats (Giridharan et al., 2022)) investigated cognitive impairment associated with astrocyte biology using heterogeneous methodologies that were also distinct from AD and PD models. Notably, five studies were in the context of high fat diet (HFD)-induced obesity (Syeda et al., 2022; Gao et al., 2020; Wu et al., 2021; Shi et al., 2020; Tian et al., 2024), one with repetitive mild TBI (Angoa-Pérez et al., 2020), one with 27-hydroxycholesterol supplementation (Hao et al., 2023), two with supplementation with the microbial metabolite trimethylamine *N*-oxide (Hoyles et al., 2021; Brunt et al., 2021) and three in the context of bacterial antigens and systemic inflammation (Giridharan et al., 2022; Zheng et al., 2023; Wei et al., 2020).

Lastly, 13 studies (9 in mice (Xu et al., 2024; Wang et al., 2023a; Zhang et al., 2019; Lakosa et al., 2023; Zhou et al., 2023; Radford-Smith et al., 2022; Wang et al., 2023b; Huang et al., 2023; Chu et al., 2019), 4 in rats (Li et al., 2021; Lv et al., 2019; Hosseinifard et al., 2019; Diviccaro et al., 2019)) assessed anxiety and depressive-like behaviours as the primary outcome. Again, methodologies to elicit such behaviours were highly variable between studies and included chronic restraint stress (CRS) (Xu et al., 2024; Zhou et al., 2023), chronic social defeat stress (CSDS) (Wang et al., 2023a), chronic unpredictable mild stress (CUMS) (Li et al., 2021; Zhang et al., 2019; Lv et al., 2019), nicotine (Lakosa et al., 2023) or finasteride (Diviccaro et al., 2019) withdrawal, HFD and streptozotocin-induced diabetes (Hosseinifard et al., 2019), fear conditioning (Chu et al., 2019), 2,2′,4,4′-tetrabromodiphenyl ether (BDE47) supplementation (Wang et al., 2023b), germ-free status (Huang et al., 2023), and maternal diet-induced obesity (Radford-Smith et al., 2022).

Across all 53 studies, only four studies (Ahnstedt et al., 2020; Qian et al., 2023; Zhou et al., 2024; Radford-Smith et al., 2022) assessed both male and females, while 42 studies (Duan et al., 2024; Li et al., 2021, 2022; Zhang et al., 2019, 2024a; Silva de Carvalho et al., 2022; Ma et al., 2019; Cuervo-Zanatta et al., 2023; Wang et al., 2019, 2022, 2023a, 2023b, 2024; Pan et al., 2022; Qu et al., 2022; Giridharan et al., 2022, 2023; Jang et al., 2020; Cui et al., 2022; Liu et al., 2022; Zhou et al., 2019b, 2021; Fang et al., 2019; Sun et al., 2018; Guo et al., 2023; Dwyer et al., 2021; Hao et al., 2023; Syeda et al., 2022; Gao et al., 2020; Zheng et al., 2023; Wei et al., 2020; Wu et al., 2021; Hoyles et al., 2021; Angoa-Pérez et al., 2020; Shi et al., 2020; Brunt et al., 2021; Tian et al., 2024; Lakosa et al., 2023; Lv et al., 2019; Chu et al., 2019; Hosseinifard et al., 2019; Diviccaro et al., 2019) assessed only males, and three studies assessed only females (Syeda et al., 2018; Xu et al., 2024; Zhou et al., 2023). Sex was not specified in four studies (Liu et al., 2020; Colombo et al., 2021; Kim et al., 2020; Huang et al., 2023).

### 3.2 Overview of astrocyte reporting methods

Brain astrocyte biology was most commonly reported as a change in the expression of GFAP, by immunohistochemical methods in 12 studies (Duan et al., 2024; Li et al., 2022; Silva de Carvalho et al., 2022; Ma et al., 2019; Wang et al., 2023b, 2024; Cui et al., 2022; Liu et al., 2022; Fang et al., 2019; Hoyles et al., 2021; Angoa-Pérez et al., 2020; Diviccaro et al., 2019), immunofluorescence in 32 studies (Zhang et al., 2019, 2024a; Ahnstedt et al., 2020; Liu et al., 2020; Syeda et al., 2018, 2022; Cuervo-Zanatta et al., 2023; Pan et al., 2022; Wang et al., 2019, 2022, 2023a; Colombo et al., 2021; Qian et al., 2023; Qu et al., 2022; Giridharan et al., 2022, 2023; Kim et al., 2020; Cui et al., 2022; Zhou et al., 2019b, 2023, 2024; Sun et al., 2018; Hao et al., 2023; Gao et al., 2020; Zheng et al., 2023; Wei et al., 2020; Wu et al., 2021; Tian et al., 2024; Li et al., 2021; Xu et al., 2024; Lakosa et al., 2023; Lv et al., 2019), Western blot (mostly in tandem with immunofluorescence) in 14 studies (Liu et al., 2020, 2022; Pan et al., 2022; Jang et al., 2020; Zhou et al., 2021, 2023, 2024; Guo et al., 2023; Dwyer et al., 2021; Wei et al., 2020; Shi et al., 2020; Brunt et al., 2021; Zhang et al., 2019; Wang et al., 2023b), and by enzyme-linked immunosorbent assay in a single study (Hosseinifard et al., 2019). Protein expression of lipocalin 2 (LCN2) as a marker of astrocyte activation was assessed in whole brain lysates in another study (Brunt et al., 2021).

Few studies attempted to assess changes in astrocyte metabolism. Assessment of brain mitochondrial translocator protein 18 kDa (TSPO) ligand binding was assessed in one study alongside GFAP immunofluorescence (Giridharan et al., 2022), and 18F-FDG PET imaging alongside GFAP/GLUT1 immunofluorescence co-localisation in another study (Hao et al., 2023). Astrocyte biology was assessed by single-nucleus transcriptomics in two studies (Huang et al., 2023; Chu et al., 2019), and qPCR of metabolic genes expressed specifically by astrocytes (*PFKFB3, ATP1A2*) alongside brain metabolomics was quantified in an additional study (Radford-Smith et al., 2022).

Regional assessment of astrocyte biology was highly variable and somewhat model-dependent across studies. The substantia nigra was investigated in 9 studies (Jang et al., 2020; Cui et al., 2022; Zhou et al., 2019b, 2021, 2024; Fang et al., 2019; Sun et al., 2018; Guo et al., 2023; Dwyer et al., 2021), the hippocampus in 24 studies (Liu et al., 2020; Syeda et al., 2018; Cuervo-Zanatta et al., 2023; Wang et al., 2019, 2023b, 2024; Pan et al., 2022; Qu et al., 2022; Giridharan et al., 2022, 2023; Hao et al., 2023; Gao et al., 2020; Zheng et al., 2023; Wei et al., 2020; Wu et al., 2021; Hoyles et al., 2021; Shi et al., 2020; Tian et al., 2024; Li et al., 2021; Xu et al., 2024; Zhang et al., 2019; Lv et al., 2019; Huang et al., 2023; Diviccaro et al., 2019), the cortex in 11 studies (Ahnstedt et al., 2020; Ma et al., 2019; Liu et al., 2020; Pan et al., 2022; Wang et al., 2019; Colombo et al., 2021; Qian et al., 2023; Qu et al., 2022; Hao et al., 2023; Gao et al., 2020; Zheng et al., 2023), and specifically the prefrontal cortex in a further 11 studies (Giridharan et al., 2022, 2023; Kim et al., 2020; Syeda et al., 2022; Xu et al., 2024; Wang et al., 2023a; Zhou et al., 2023; Radford-Smith et al., 2022; Huang et al., 2023; Chu et al., 2019; Hosseinifard et al., 2019). Less commonly studied were the amygdala (Hosseinifard et al., 2019), caudate nucleus (Li et al., 2022), entorhinal cortex (Syeda et al., 2018; Cuervo-Zanatta et al., 2023; Hoyles et al., 2021), hypothalamus (Wang et al., 2023b), optic tract (Angoa-Pérez et al., 2020), striatum (Silva de Carvalho et al., 2022; Ahnstedt et al., 2020; Jang et al., 2020; Wang et al., 2022), thalamus (Ahnstedt et al., 2020), and the ventral tegmental area (Lakosa et al., 2023) and nucleus accumbens (Lakosa et al., 2023). Whole brain lysate used in two studies (Duan et al., 2024; Brunt et al., 2021) and the brain region examined was not specified in two studies (Zhang et al., 2024a; Liu et al., 2022).

### 3.3 Overview of the gut microbiota

Gut microbiota from faeces were analysed mostly using 16S rRNA sequencing methods. One study employed metagenomic sequencing (Wang et al., 2023a), and another utilised targeted qPCR to quantify bacteria (Radford-Smith et al., 2022). Short-chain fatty acids (SCFAs) were analysed by targeted gas chromatography mass spectrometry in 12 studies (Duan et al., 2024; Ahnstedt et al., 2020; Syeda et al., 2018; Cuervo-Zanatta et al., 2023; Colombo et al., 2021; Giridharan et al., 2022, 2023; Wang et al., 2022; Sun et al., 2018; Gao et al., 2020; Shi et al., 2020; Tian et al., 2024), while five studies probed faecal metabolites using untargeted metabolomic methods (Pan et al., 2022; Zhou et al., 2019b; Radford-Smith et al., 2022; Wang et al., 2023b; Chu et al., 2019). Lastly, three studies specifically investigated the microbial metabolite trimethylamine N-oxide (TMAO) (Li et al., 2022; Hoyles et al., 2021; Brunt et al., 2021).

### 3.4 Interventions

The majority of studies utilised at least one additional intervention to modify disease pathology in the context of the gut microbiota-astrocyte axis. This included treatment with a specific probiotic (Ma et al., 2019; Fang et al., 2019; Dwyer et al., 2021; Xu et al., 2024; Wang et al., 2023a; Zhou et al., 2023; Radford-Smith et al., 2022; Hosseinifard et al., 2019), prebiotic (Cuervo-Zanatta et al., 2023; Gao et al., 2020; Shi et al., 2020), a form of microbial depletion and/or reconstitution (Qian et al., 2023; Kim et al., 2020; Cui et al., 2022; Zhou et al., 2019b, 2023, 2024; Sun et al., 2018; Wang et al., 2023a; Zhang et al., 2019; Lakosa et al., 2023; Lv et al., 2019; Huang et al., 2023; Chu et al., 2019), or a comparison of germ free and specific pathogen free animals without microbial reconstitution (Colombo et al., 2021; Huang et al., 2023; Chu et al., 2019). Other studies assessed the effect of a specific drug, metabolite, or other bioactive compound on neuropathology (Duan et al., 2024; Li et al., 2022; Zhang et al., 2024a; Liu et al., 2020, 2022; Syeda et al., 2018, 2022; Wang et al., 2019, 2022, 2024; Colombo et al., 2021; Qu et al., 2022; Cui et al., 2022; Zhou et al., 2021; Guo et al., 2023; Hao et al., 2023; Wu et al., 2021; Tian et al., 2024; Diviccaro et al., 2019), or fasting/restrictive diets (Silva de Carvalho et al., 2022; Pan et al., 2022; Zhou et al., 2019b), sepsis (Giridharan et al., 2023), or acupuncture (Jang et al., 2020; Li et al., 2021). Eight of the studies did not contain an additional intervention (Ahnstedt et al., 2020; Giridharan et al., 2022; Zheng et al., 2023; Wei et al., 2020; Hoyles et al., 2021; Angoa-Pérez et al., 2020; Brunt et al., 2021; Wang et al., 2023b).

### 3.5 Outcomes

**Acute brain injury:** Stroke and acute TBI were associated with an increase in brain GFAP expression (SMD 11.24 [4.08–18.40], Fig. 2) alongside motor impairment compared to sham controls (Duan et al., 2024; Zhang et al., 2024a; Silva de Carvalho et al., 2022; Ahnstedt et al., 2020; Ma et al., 2019). A number of studies noted increased gut (Zhang et al., 2024a; Ahnstedt et al., 2020; Ma et al., 2019) and brain (Zhang et al., 2024a; Silva de Carvalho et al., 2022; Ahnstedt et al., 2020; Ma et al., 2019) permeability post-infarction through increases in TLR4 expression or reduced expression of tight junction proteins (occludin and ZO-1). Amelioration of permeability was linked to reduced neuroinflammation (reduction in lesion or infarct size, and/or reduced perilesional tumour necrosis factor [TNF] and interleukin [IL]-1B expression) and improved behavioural outcomes through a mediating role of the gut microbiota, which was attributed to the augmentation by faecal microbiota transplant (FMT) (Duan et al., 2024; Zhang et al., 2024a), butyrate (Duan et al., 2024), or post-injury probiotic treatment (Ma et al., 2019). Similarly, loss of butyrate via butyrate-producing bacteria post-stroke was associated with increased astrocyte activation and poorer outcomes (Ahnstedt et al., 2020), as was an increased circulating level of microbial metabolite TMAO (Li et al., 2022).

**Fig. 2:**
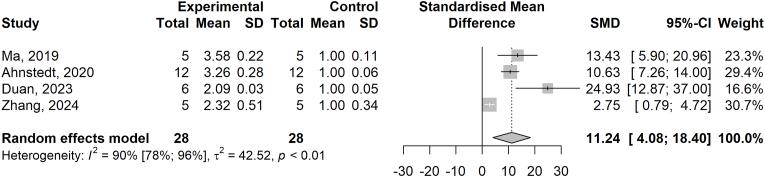
Forest plot of GFAP expression in rodent models of acute brain injury (ABI). The forest plot displays the standardized mean differences (SMD) in GFAP expression between control and disease groups across studies. Each study is represented by a square, with the size proportional to the study's weight in the meta-analysis. Horizontal lines represent 95 % confidence intervals (CIs). The diamond at the bottom represents the pooled effect size, with the width indicating the 95 % CI. The dashed vertical line denotes the null effect (SMD = 0). Heterogeneity statistics (I^2^) and p-values are included. Random effects model was used.

**Fig. 3:**
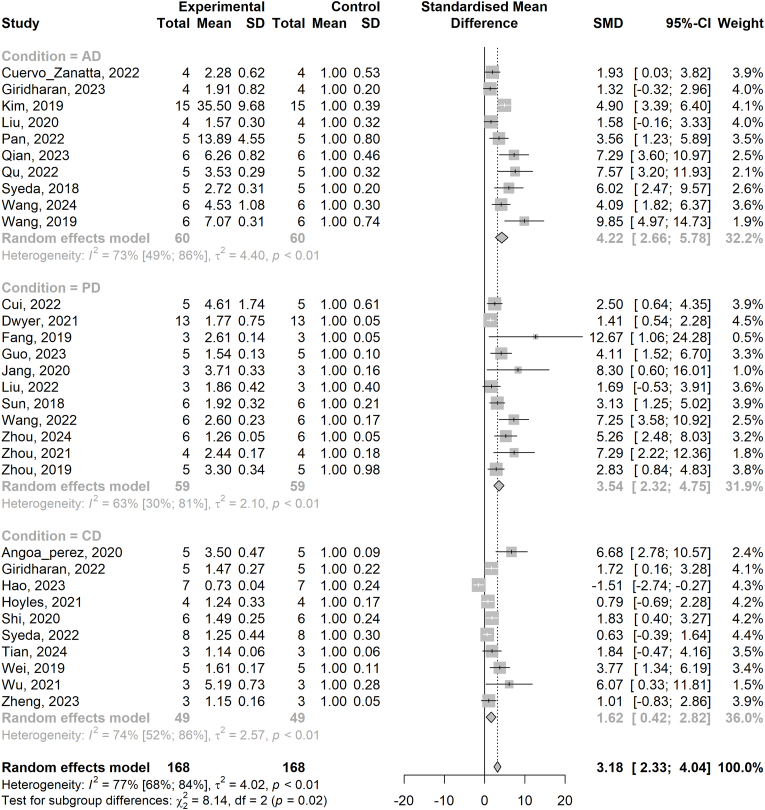
Forest plot of GFAP expression in rodent models of neurodegenerative disease. The forest plot displays the standardized mean differences (SMD) in GFAP expression between control and disease groups across studies. Each study is represented by a square, with the size proportional to the study's weight in the meta-analysis. Horizontal lines represent 95 % confidence intervals (CIs). The diamond at the bottom represents the pooled effect size, with the width indicating the 95 % CI. The dashed vertical line denotes the null effect (SMD = 0). Heterogeneity statistics (I^2^) and p-values are included. Random effects model was used. Subgroups: AD (Alzheimer's Disease), PD (Parkinson's Disease), CD (other cognitive decline).

**Neurodegeneration:** 10 AD models quantified GFAP expression (WB, IHC, or IF) as a marker of astrocyte activation (Liu et al., 2020; Syeda et al., 2018; Cuervo-Zanatta et al., 2023; Wang et al., 2019, 2024; Pan et al., 2022; Qian et al., 2023; Qu et al., 2022; Giridharan et al., 2023; Kim et al., 2020). Compared to WT controls, GFAP expression was significantly increased (SMD 4.22 [2.66–5.78], Fig. 3), which coincided with memory and learning deficits in the Morris water maze (Liu et al., 2020; Cuervo-Zanatta et al., 2023; Wang et al., 2019, 2024; Pan et al., 2022; Qian et al., 2023; Qu et al., 2022), novel object recognition test (Cuervo-Zanatta et al., 2023; Pan et al., 2022; Giridharan et al., 2023), Y/T-maze (Syeda et al., 2018; Cuervo-Zanatta et al., 2023; Kim et al., 2020), and Barnes maze (Colombo et al., 2021). In one study, the gut microbiota and associated SCFAs were identified as important *contributors* to AD pathology (Colombo et al., 2021), with reduced amyloid burden evident in germ free (GF) animals. Conversely, microbial modification in AD was also shown to ameliorate markers of neuroinflammation (e.g. reduced pro-inflammatory cytokine expression, IBA-1 expression), neuropathology (reduced amyloid-β plaque load), and cognitive deficits (Liu et al., 2020; Syeda et al., 2018; Cuervo-Zanatta et al., 2023; Wang et al., 2019, 2024; Pan et al., 2022; Qu et al., 2022; Kim et al., 2020), associated with increased gut butyrate production (Cuervo-Zanatta et al., 2023), and reduced gut permeability (Syeda et al., 2018; Kim et al., 2020). AD was associated with a loss of beneficial (e.g. SCFA-producing) microbes (Syeda et al., 2018; Wang et al., 2019; Qu et al., 2022), and other studies showed prominent dysbiosis in AD animals across the taxonomic hierarchy, most prominently an increase in the Bacteroidetes/Firmicutes ratio in AD in six independent studies utilising six different AD models (Syeda et al., 2018; Wang et al., 2024; Pan et al., 2022; Qu et al., 2022; Giridharan et al., 2023; Kim et al., 2020) (no difference in two studies (Liu et al., 2020; Cuervo-Zanatta et al., 2023), and a decreased ratio found in one study (Wang et al., 2019)).

All 11 PD models quantified GFAP expression (WB, IHC, or IF), which was also increased compared to controls (Jang et al., 2020; Cui et al., 2022; Liu et al., 2022; Wang et al., 2022; Zhou et al., 2019b, 2021, 2024; Fang et al., 2019; Sun et al., 2018; Guo et al., 2023; Dwyer et al., 2021) (SMD 3.54 [2.32–4.75], Fig. 3) and coincided with motor deficits using the pole (Cui et al., 2022; Liu et al., 2022; Wang et al., 2022; Zhou et al., 2019b, 2021, 2024; Fang et al., 2019; Sun et al., 2018; Guo et al., 2023) or rotarod (Jang et al., 2020; Dwyer et al., 2021) tests. Seven studies associated PD with reduced gut barrier integrity via reduced expression of tight junction proteins (Jang et al., 2020; Wang et al., 2022; Zhou et al., 2021, 2024; Sun et al., 2018; Guo et al., 2023; Dwyer et al., 2021) and one with reduced blood-brain barrier integrity (Liu et al., 2022). PD was associated with increased SCFAs in three studies (Wang et al., 2022; Zhou et al., 2019b; Sun et al., 2018) and pathology improved by antibiotic depletion of the microbiome in a further study (Zhou et al., 2024). Conversely, PD pathology was ameliorated by microbial modification and increased SCFAs, especially butyrate (Guo et al., 2023), in four studies (Jang et al., 2020; Liu et al., 2022; Zhou et al., 2021; Guo et al., 2023). Notably, phylum Bacteroidetes/Firmicutes ratio was again increased in PD in five studies (Jang et al., 2020; Liu et al., 2022; Wang et al., 2022; Sun et al., 2018; Guo et al., 2023) and decreased in one study (Cui et al., 2022). One study showed no effect of PD on microbiota composition, which was an atypical PD model of LPS and paraquat injection (Dwyer et al., 2021).

GFAP expression (IF, IHC, or WB) was quantified in 10 (non-AD/non-PD) models of cognitive impairment (SMD 1.62 [0.42–2.82], Fig. 3) (Hao et al., 2023; Giridharan et al., 2022; Syeda et al., 2022; Zheng et al., 2023; Wei et al., 2020; Wu et al., 2021; Hoyles et al., 2021; Angoa-Pérez et al., 2020; Shi et al., 2020; Tian et al., 2024), with a further study quantifying LCN2 as a measure of astrocyte activation (1.67x increase following 6-months TMAO supplementation) (Brunt et al., 2021). In addition to GFAP, TSPO binding increased proportional to increased GFAP expression secondary to sepsis (Giridharan et al., 2022) and ^18^F-FDG PET imaging showed decreased uptake in line with reduced GFAP expression secondary to 27-hydroxycholesterol-induced cognitive impairment (Hao et al., 2023). Of the five studies addressing cognitive decline in the context of high fat diet (HFD)-induced obesity (Syeda et al., 2022; Gao et al., 2020; Wu et al., 2021; Shi et al., 2020; Tian et al., 2024), chronic HFD consumption consistently reduced gut barrier integrity (Syeda et al., 2022; Gao et al., 2020; Wu et al., 2021; Shi et al., 2020; Tian et al., 2024). Prebiotic intake in particular was linked to an increase in SCFAs, which may have mediated subsequent amelioration of gut (and blood-brain (Shi et al., 2020)) barrier integrity and reduced cognitive impairment (Gao et al., 2020; Shi et al., 2020; Tian et al., 2024). In other studies, inflammatory challenges were associated with an increased gut Bacteroides/Firmicutes ratio (Hao et al., 2023; Zheng et al., 2023), reduced gut and blood-brain barrier integrity (Zheng et al., 2023), and reduced gut SCFA levels (Giridharan et al., 2022), the latter demonstrating a direct negative correlation between faecal butyrate levels and PFC and hippocampal TSPO expression (Giridharan et al., 2022).

Overall, GFAP expression was increased in 31 rodent models of neurodegeneration (total n per group = 168), with a SMD of 3.18 [2.33–4.04]. Whilst AD models demonstrated the greatest increase in GFAP expression compared to their respective controls, and CD models the smallest increase, the SMD was not significantly different overall between subgroups (p = 0.02, Fig. 3).

**Fig. 4:**
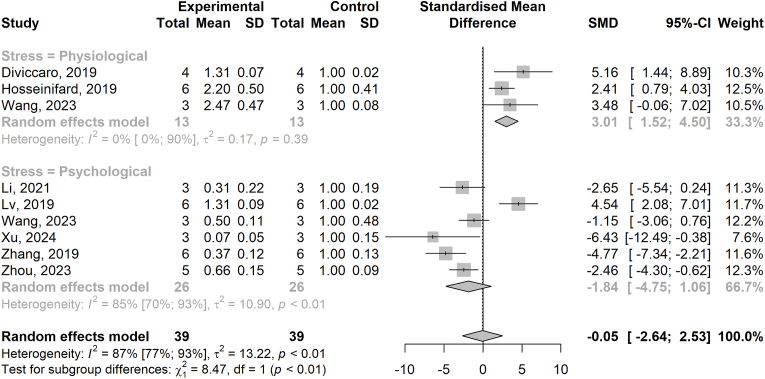
Forest plot of GFAP expression in rodent models of mood disorder. The forest plot displays the standardized mean differences (SMD) in GFAP expression between control and disease groups across studies. Each study is represented by a square, with the size proportional to the study's weight in the meta-analysis. Horizontal lines represent 95 % confidence intervals (CIs). The diamond at the bottom represents the pooled effect size, with the width indicating the 95 % CI. The dashed vertical line denotes the null effect (SMD = 0). Heterogeneity statistics (I^2^) and p-values are included. Random effects model was used. Subgroups: physiological and psychological stress.

**Mood disorder:** Brain GFAP expression was quantified in 9 of 13 studies addressing the MGA axis in anxiety and depressive-like behaviour (ELISA, IF, IHC, or WB). GFAP expression was reduced in five chronic stress models compared to controls (Li et al., 2021; Xu et al., 2024; Wang et al., 2023a; Zhang et al., 2019; Zhou et al., 2023) and was increased in one study of CUMS (Lv et al., 2019), BDE47 supplementation (Wang et al., 2023b), diabetes (Hosseinifard et al., 2019), and finasteride withdrawal, an inhibitor of the enzyme 5α-reductase (Diviccaro et al., 2019). Upon meta-analysis, physiological stress (Wang et al., 2023b; Hosseinifard et al., 2019; Diviccaro et al., 2019) leading to depressive and anxiety-like behaviours led to a significant increase in GFAP expression (SMD 3.01 [1.52–4.50], Fig. 4) whereas psychological stress models of mood dysfunction numerically reduced GFAP expression but did not reach statistical significance (SMD -1.84 [−4.75-1.06], Fig. 4). The overall SMD of GFAP expression in models of mood disorder was not significant at SMD -0.05 [−2.64-2.53] (Fig. 4) with high study heterogeneity (*I*^*2*^ = 87 %) and clear subgroup differences (p < 0.01). Nevertheless, both increases and decreases in GFAP expression seemed to be related to increased depressive-like behaviours in either the tail suspension test, forced-swim test, or sucrose preference test, indicating a complex relationship between stress, astrocyte reactivity, and behaviour. GFAP expression was also found to be reduced by antibiotic depletion of gut microbiota independent of depressive-like behaviours (Lakosa et al., 2023), and the expression of astrocyte-specific metabolic gene *PFKFB3* was increased following early-life probiotic exposure (Radford-Smith et al., 2022). Additionally, *PFKFB3* expression in the PFC was significantly correlated with faecal butyrate levels, suggesting a potential mediatory role of the gut microbiota and associated SCFAs on the mediatory role between probiotic intake and reduced depressive and anxiety-like behaviours induced by exposure to maternal obesity (Radford-Smith et al., 2022). Two studies employed single-nucleus transcriptomics of antibiotic-depleted or GF vs. specific pathogen-free (SPF) animals, enabling high-resolution analysis of astrocyte function in the context of impaired fear extinction (Chu et al., 2019) and reduced anhedonia (Huang et al., 2023), respectively. Both studies identified a significant number of differentially-expressed genes (DEGs) in astrocytes in ABX-depleted or GF mice compared to SPF controls. Notably, transcriptomically distinct astrocyte subsets both expressed GFAP, highlighting the functional diversity of GFAP + cells in the brain (Chu et al., 2019). Depressive-like behaviour was associated with increased faecal Bacteroidetes/Firmicutes ratio in four studies (Li et al., 2021; Zhang et al., 2019; Hosseinifard et al., 2019; Diviccaro et al., 2019), and reduced gut barrier integrity in four studies (Xu et al., 2024; Zhou et al., 2023; Wang et al., 2023b; Lv et al., 2019). Resultant increases in endotoxemia and systemic inflammation were related to altered tryptophan metabolism and reduced neuroprotective kynurenic acid production in two studies (related to reduced GFAP expression following chronic stress) (Xu et al., 2024; Zhou et al., 2023). Reduced microbiome-derived ammonia synthesis following chronic stress was also shown as a key rate-limiting step in astrocyte glutamine production linked to poor resilience in mice (Wang et al., 2023a).

## 4 Discussion

**Fig. 5:**
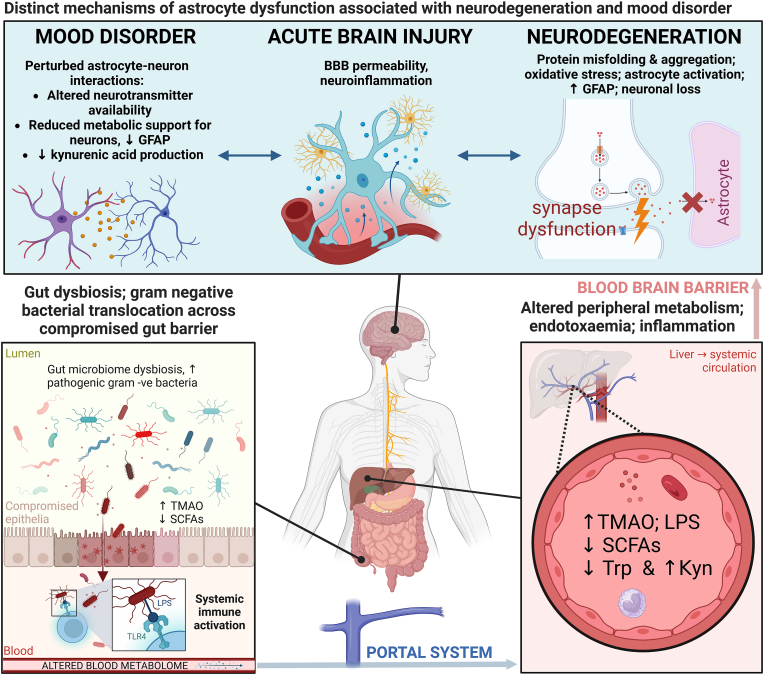
Overview of convergent mechanisms of microbiota-gut-astrocyte axis perturbation in models of acute brain injury, neurodegeneration, and mood disorder as determined by systematic review of *in vivo* studies in rodents. BBB, blood-brain barrier; GFAP, glial fibrillary acidic protein; Kyn, kynurenine; LPS, lipopolysaccharide; SCFAs, short-chain fatty acids; TLR4, toll-like receptor 4; TMAO, trimethylamine N-oxide; Trp, tryptophan.

This systematic review included 53 original research articles pertaining to the MGA axis *in vivo*. Full-text analysis allowed subsequent categorisation of studies into assessment of the MGA axis in acute brain injury, neurodegeneration, and mood disorders. 44 of 53 studies were eligible for a meta-analysis of GFAP expression (Table S2). Whereas GFAP expression tended to be elevated in rodent models of acute brain injury and neurodegeneration, especially AD and PD, GFAP expression was suppressed in five studies of stress-induced mood disorder, and increased in four studies inducing physiological stress. Overall, this suggests that changes to astrocyte biology in mood disorder could be characterised by either, or both, a reactive, GFAP + phenotype or primarily by a loss of homeostatic function (Patani et al., 2023), whereas local injury and inflammatory insult is more consistently associated with reactive astrogliosis (Carter et al., 2019) (Fig. 5).

Surprisingly, despite the variation in GFAP expression between disease models, common mechanisms of MGA axis perturbation were observed across distinct neuropathology. Most notably, reduced gut barrier integrity was identified in three models of acute brain injury (Zhang et al., 2024a; Ahnstedt et al., 2020; Ma et al., 2019), three AD models (Syeda et al., 2018; Cuervo-Zanatta et al., 2023; Kim et al., 2020), seven PD models (Jang et al., 2020; Wang et al., 2022; Zhou et al., 2021, 2024; Sun et al., 2018; Guo et al., 2023; Dwyer et al., 2021), five models of obesity-induced cognitive impairment (Syeda et al., 2022; Gao et al., 2020; Wu et al., 2021; Shi et al., 2020; Tian et al., 2024), inflammation-associated cognitive impairment (Zheng et al., 2023), and four models of depressive-like behaviour (Xu et al., 2024; Zhou et al., 2023; Wang et al., 2023b; Lv et al., 2019) (Fig. 5).

An increased Bacteroidetes/Firmicutes ratio was also identified in 17 studies across the neurodegenerative (Syeda et al., 2018; Wang et al., 2022, 2024; Pan et al., 2022; Qu et al., 2022; Giridharan et al., 2023; Kim et al., 2020; Jang et al., 2020; Liu et al., 2022; Sun et al., 2018; Guo et al., 2023; Hao et al., 2023; Zheng et al., 2023) and the mood disorder (Li et al., 2021; Zhang et al., 2019; Hosseinifard et al., 2019; Diviccaro et al., 2019) studies. An increase in Bacteroidetes/Firmicutes is also consistent with AD microbiota changes in humans (Vogt et al., 2017), but this is the first review to identify a pattern of microbial shift between neurodegenerative and mood disorders. Bacteroidetes comprise three classes of gram-negative bacteria and it is therefore unsurprising that this pattern of gut dysbiosis was associated with increased plasma lipopolysaccharide levels and expression of gut, as well as brain, TLR4 (Wang et al., 2022; Sun et al., 2018), and reduced BBB integrity (Liu et al., 2022; Zheng et al., 2023; Shi et al., 2020).

Studies in the last decade have also explored ways in which the microbiome can be manipulated to promote BBB integrity. Strategies to reverse gut microbiome dysbiosis following neurological insult are promising therapeutic targets. For example, reversing dysbiosis through the ingestion notoginseng saponins restored BBB integrity via increased occludin and Zo1 expression, and reduced proinflammatory cytokine expression (IL-1B, IL-6, TNF) in a rodent model of ischemic stroke (Zhang et al., 2024a). The effect was shown to be microbiota-dependent, as the therapeutic effects were recapitulated through FMT of notoginseng-treated animals. Notoginseng has been shown to reduce behavioural despair in rodent models of depression through similar mechanisms (Kim and Cho, 2021), further illustrating the utility of understanding and targeting common pathophysiological mechanisms. The efficacy of Notoginseng saponins in the treatment of ischemic stroke is now being tested in patients with ischemic stroke, with positive initial results on stroke outcomes (increased likelihood of functional independence 3-months post-stroke) (Wu et al., 2023). This translational study underscores the potential of microbiota manipulation as a promising target for neurological disease treatment, though faecal microbiota analysis was not reported. Further translational research is needed to elucidate and confirm the contribution of GMA perturbation to neurological disease in humans, with the goal of optimising therapeutic strategies for clinical application.

While these examples highlight the therapeutic promise of manipulating the gut microbiota to improve outcomes across neurological diseases, it is important to acknowledge limitations in our current understanding. The common microbiota disruptions identified across disease models, such as increases in the Bacteroidetes/Firmicutes ratio or reduced SCFA production, were largely observed at the phylum level, which provides limited resolution and may obscure more granular, disease-specific changes at the genus or species level. Likewise, although gut barrier disruption and SCFA depletion are recurring features, the downstream effects of these perturbations are likely to interact in complex ways with disease-specific processes, including neuroinflammatory pathways, protein aggregation, or neuronal vulnerability, that may not be fully recapitulated in rodent models. Thus, although interventions targeting gut integrity and microbial balance (e.g. prebiotic fibres, SCFA supplementation, dietary modulation) may offer broad therapeutic potential, stratification based on disease phenotype, microbial profiles, and host responses will be critical to achieve precision and efficacy in clinical translation (Loh et al., 2024; Hediyal et al., 2024). Future research should focus on disentangling shared versus disease-specific microbiota–astrocyte pathways to inform the rational design of targeted microbiota-directed interventions (Loh et al., 2024; Hitch et al., 2022). The similarities between the findings in the AD and PD models, as well as models of depressive-like behaviour, is intriguing and strongly argues for a role of the Bacteroidetes/Firmicutes ratio in shared astrocyte dyshomeostasis, but it also raises the ‘streetlight’ issue as researchers then flock to such outcome measures after they have been identified, which can leave us in ignorance of other, potentially, more important associations.

The mechanisms underlying how gut microbiota perturbations influence astrocyte biology remain incompletely understood. However, existing studies suggest several plausible pathways: increased exposure of the CNS to microbial products, particularly lipopolysaccharides (LPS), via compromised gut and blood-brain barrier integrity; altered microbial metabolite production, including short-chain fatty acids (SCFAs), which directly influence astrocyte metabolism and inflammation; and disrupted tryptophan metabolism leading to altered serotonergic signalling and neuroinflammation. Current evidence, primarily from faecal microbiota transplantation (FMT) and 10.13039/100023195SCFA supplementation studies, strongly supports these causal links. For instance, FMT from healthy rodents ameliorated pathological increases in GFAP expression in diseased animals, likely through restoration of gut and BBB integrity and reduced neuroinflammation (Duan et al., 2024; Zhang et al., 2024a; Qian et al., 2023; Kim et al., 2020; Zhou et al., 2021, 2023; Sun et al., 2018). Conversely, transplanting microbiota from diseased animals into naïve rodents induced astrocyte pathology (Zheng et al., 2023; Lv et al., 2019). These findings provide compelling evidence for the microbiota's active role in modulating astrocyte function, potentially mediated by microbial toxins such as LPS and subsequent inflammatory signalling in astrocytes and microglia (Robb et al., 2020). However, the precise molecular and cellular mechanisms require further elucidation through advanced approaches like astrocyte-specific metabolic tracing and transcriptomics.

While these findings highlight the potential of FMT as a powerful experimental tool to establish causality in microbiota–astrocyte interactions, its translation as a therapeutic strategy in neurological disorders remains challenging. Variability in donor microbiota composition, lack of standardised transplantation protocols, and incomplete understanding of which microbial taxa or metabolites mediate beneficial effects introduce considerable uncertainty (Hediyal et al., 2024). Moreover, neurological conditions are heterogeneous and it remains unclear whether a universal donor approach will be effective across diseases or whether disease-specific or personalised microbial consortia will be required (Settanni et al., 2021). Future research should aim to deconstruct FMT into defined microbial communities or metabolite-based interventions, which may offer enhanced reproducibility, mechanistic clarity, and greater safety for clinical application (Zhang et al., 2024b).

This review describes how the presence of gut microbiota-derived metabolites, including short-chain fatty acids (SCFAs) especially, in the context of neuroinflammation, appear to be correlated with astrocyte activation. It seems likely that the presence of SCFAs may contribute to the restoration of astrocyte function, whereas their absence may exacerbate pathological conditions (Fig. 5). This dual role of SCFAs as effector molecules of the gut microbiome highlights their potential in either exacerbating or resolving pathology. However, the overarching conclusion from our examination of the literature is the importance of understanding astrocyte metabolic pathways in the context of gut microbiota interactions.

Currently, there is limited information on the impact of the gut microbiome on the regional anatomical behaviour of protoplasmic astrocytes (and their subtypes) or fibrous astrocytes. It appears unlikely that these changes are global, or that all astrocyte behaviours are conserved across distinct pathologies or mood disorders. However, the observed commonalities are encouraging and suggest potential avenues for exploring new therapies in the future. To further understand the contribution of astrocytes to disease states, studies employing high-resolution spatial transcriptomic and proteomic single-cell assays of astrocyte function are essential. These advanced techniques will provide deeper insights into the specific roles of astrocytes in neuroinflammation and their interactions with the gut microbiome, potentially leading to novel therapeutic strategies.

Emerging single-cell and single-nucleus transcriptomic studies included in this review suggest that astrocyte subtypes may respond differently to gut microbiota perturbations. For example, microbiota depletion altered gene expression in regionally distinct astrocyte populations, including downregulated of *Slc4a4* (Chu et al., 2019) (sodium-bicarbonate cotransporter 1) in mPFC astrocytes*.* Reduced astrocytic *Slc4a4* expression may regionally impair BBB integrity (Ye et al., 2024). In another study, GF mice showed reduced microglia-astrocyte communication in the hippocamus, specifically in *Adora1*-expressing astrocytes and CD39^+^ microglia (Huang et al., 2023). This specific interaction is thought to be crucial for limiting excessive neuronal activity, similar to inhibitory neurons, in both healthy and disease states (Badimon et al., 2020). It remains challenging to capture the full extent of astrocyte heterogeneity, as current single-cell and spatial transcriptomic approaches often lack sufficient power to detect rare sub-states or to distinguish dynamic and transient reactive phenotypes across disease stages (Patani et al., 2023). Moreover, most available datasets provide only partial insight into baseline regional heterogeneity, and far less into within-disease variability or how astrocyte subtypes might differ between distinct neurological conditions (Patani et al., 2023). As the field advances, more comprehensive and properly powered single-cell and spatial multi-omics approaches, combined with metabolic tracing and fate-mapping tools, will be critical to resolve these limitations and to identify disease-relevant astrocyte states. Only with such high-resolution data will targeted therapeutic strategies tailored to astrocyte dysfunction across different neurological disorders become feasible. We recognise the limitations of this review, and the data reported in each study. GFAP expression, the mostly widely reported molecular readout of astrocyte biology across studies, provides a limited insight into astrocyte behaviour, and future MGA axis studies and reviews must incorporate more comprehensive phenotyping of astrocytes. A modest publication bias was detected regarding GFAP expression. However, given both increased and decreased GFAP expression is considered a feature of mood disorder, and that in general, GFAP expression was a minor outcome of each individual study, it is unlikely to effect the interpretation of our findings. We also identified a high degree of variability in the animal models of Alzheimer's Disease, cognitive decline, and mood disorder, which may contribute to increased statistical heterogeneity in these subgroups.

Given the methodological heterogeneity observed across studies, we employed random-effects meta-analysis stratified by disease class to accommodate variability. Outliers and missing data were managed conservatively; studies lacking clear control groups or containing insufficiently detailed data extraction were excluded from the quantitative synthesis. While random-effects modelling accounts partially for heterogeneity, future analyses will benefit from employing more sophisticated statistical methods, including multilevel models or meta-regression, particularly as the dataset expands and standardisation across studies improves.

It is worth noting that there are limitations inherent to several of the rodent models included in this review. For example, while MPTP-induced Parkinsonism and intracranial toxin injection models produce reproducible motor deficits and glial activation, they lack key features of human Parkinson's disease, including progressive dopaminergic neuronal loss in the substantia nigra and the hallmark presence of Lewy bodies, thus limiting their construct validity. Likewise, models of mood disorders—such as chronic stress paradigms—may not replicate the full complexity of human depression but they often demonstrate predictive validity, notably via responsiveness to antidepressant drugs including SSRIs. These models, despite their limitations, remain widely used due to their reproducibility and utility in identifying putative biological mechanisms. Furthermore, the inclusion of acupuncture studies warrants caution: while some report beneficial effects on gut–brain axis parameters, the absence of a placebo effect in rodents raises questions regarding translatability, given that placebo responses are likely central to clinical acupuncture efficacy in humans. These caveats highlight the need for careful interpretation and triangulation of preclinical findings with human studies.

Additionally, despite significant study heterogeneity, subgroup analysis revealed notable differences in GFAP expression linked to the nature of stress paradigms used to induce depressive-like behaviour. Specifically, physiological stress models (e.g., chemical, drug withdrawal, and metabolic stressors) significantly increased GFAP expression, which was linked to peripheral inflammation, systemic endotoxaemia, and barrier disruption ultimately resulting in raised brain inflammatory markers such as IL-6 (Wang et al., 2023b; Hosseinifard et al., 2019) and TNF (Diviccaro et al., 2019). In contrast, psychological stress models (e.g., chronic unpredictable mild stress, chronic restraint stress) typically resulted in reduced GFAP expression, suggestive of astrocyte dyshomeostasis. These effects may reflect impaired serotonergic signalling (Jang et al., 2020; Zhou et al., 2023) and associated increases in the tryptophan–kynurenine pathway (Xu et al., 2024; Zhou et al., 2023), as well as reduced gut ammonia production resulting in GABAergic deficits (Wang et al., 2023a), all of which are known to modulate astrocyte function (Sidoryk-Węgrzynowicz et al., 2024; Minhas et al., 2024). Notably, gut-barrier disruption was a consistent feature across both physiological (Wang et al., 2023b) and psychological (Xu et al., 2024; Zhou et al., 2023; Lv et al., 2019) stress models, as was an increase in the gut Bacteroidetes/Firmicutes ratio (Li et al., 2021; Zhang et al., 2019; Hosseinifard et al., 2019; Diviccaro et al., 2019). This divergence underscores the complexity of astrocyte responses to stress and mirrors the clinical heterogeneity of human depression. When translating these findings, future studies and clinical trials targeting the MGA axis should incorporate both mechanistic understanding and patient stratification strategies to ensure alignment between preclinical models and the diverse pathophysiological subtypes of depression observed in the clinic.

Future research in this area must address the noticeable bias towards male rodent models. Only four of 53 studies investigated both males and females, and just three exclusively studied females, compared to 42 studies that solely used male rodents (sex not specified in four studies). This bias is problematic, given reported sex-specific differences in immune and metabolic responses to microbiota modulation (Radford-Smith et al., 2022; Saha and Sisodia, 2024; Gozlan et al., 2024), potentially influencing astrocyte reactivity and neuroinflammation (Zhou et al., 2024). For instance, probiotic interventions exhibit sex-dependent efficacy in models of Parkinson's disease, highlighting the necessity of including both sexes to enhance translational relevance.

Moreover, the majority of studies included young adult rodents, whereas many neurological disorders predominately affect older populations. This age discrepancy significantly limits generalisability, as immune function (Montecino-Rodriguez et al., 2013), astrocyte reactivity (Gildea and Liddelow, 2025), and gut microbiota composition (Bradley and Haran, 2024) exhibit considerable age-dependent changes. Future studies must therefore systematically investigate older animals, especially given the ageing population and the age-dependent progression of human neurological diseases.

In addition, the role of the MGA axis in other neurodegenerative disorders warrants exploration. Astrocyte-mediated neuronal toxicity is a prominent feature of motor neurone disease (amyotrophic lateral sclerosis) pathophysiology (Meyer et al., 2014), and disease severity is also known to be associated with distinct commensal bacteria in preclinical studies (Blacher et al., 2019). Future work must determine whether astrocytes mediate the interplay between genetic and environmental (ie. microbiome-related) susceptibility, for example through microbial metabolite temperance of CNS neuroinflammation via stimulation of the aryl hydrocarbon receptor (Rothhammer et al., 2016). Similarly, in Huntington's disease, an increase in the Bacteroidetes/Firmicutes ratio has been observed in preclinical models of the disease (Kong et al., 2020), and dietary fibre supplementation (Gubert et al., 2024) and FMT (Gubert et al., 2022) have been shown to improve behavioural outcomes. The latter study demonstrated increased plasma acetate in FMT-treated mice with Huntington's. While acetate is thought to be important in mitigating cognitive impairment and neuroinflammation by facilitating astrocyte metabolism (including key anaplerotic reactions (He et al., 2024)), markers of astrocyte activity were not reported.

In conclusion, this systematic review highlights a surprising, common mechanism of microbiota-gut-brain axis perturbation implicated across distinct neurological diseases, including acute brain injury, neurodegeneration, and mood disorder. While the role of astrocytes in this axis has been hitherto largely overlooked, it is now emerging as a critical area of research. In anticipation of the rapid growth in this research area, we suggest that future studies move beyond GFAP expression to incorporate higher resolution of astrocyte function, including the investigation of important metabolic pathways, single-cell sequencing, and spatial delineation of astrocyte activity (Patani et al., 2023; Brandebura et al., 2023). Specifically, future research should prioritise: (i) single-cell and spatial transcriptomics to identify astrocyte subtype-specific responses to microbiota manipulation, which could reveal previously unrecognised heterogeneity in astrocyte responses; (ii) integrating functional microbial metabolomics and metagenomics alongside taxonomic profiling to clarify causal microbial metabolites driving astrocyte modulation; and (iii) standardised behavioural assessments with rigorous inclusion of sex and age variables to enhance translational applicability and address existing biases. These approaches will help pave the way for innovative therapeutic strategies to help treat neurological disease.

## CRediT authorship contribution statement

**Daniel E. Radford-Smith:** Conceptualization, Data curation, Formal analysis, Investigation, Methodology, Project administration, Writing – original draft, Writing – review & editing. **Katharine Oke:** Data curation. **Carolina F.F.A. Costa:** Data curation. **Daniel C. Anthony:** Conceptualization, Supervision, Writing – review & editing.

## Funding information

This research did not receive any specific grant from funding agencies in the public, commercial, or not-for-profit sectors.

## Declaration of competing interest

The authors declare that they have no known competing financial interests or personal relationships that could have appeared to influence the work reported in this paper.

## Figures and Tables

**figs1:**
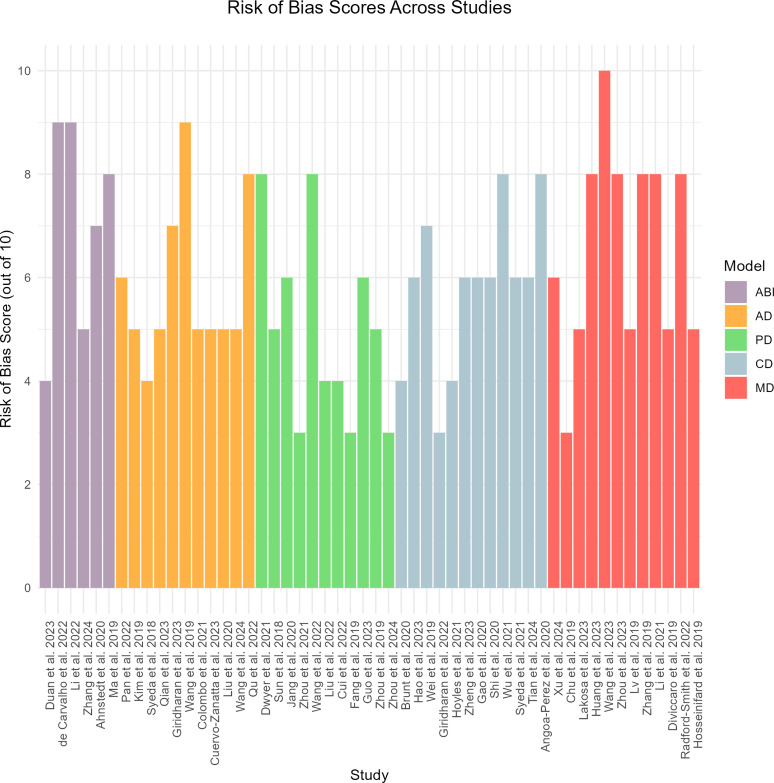


**figs2:**
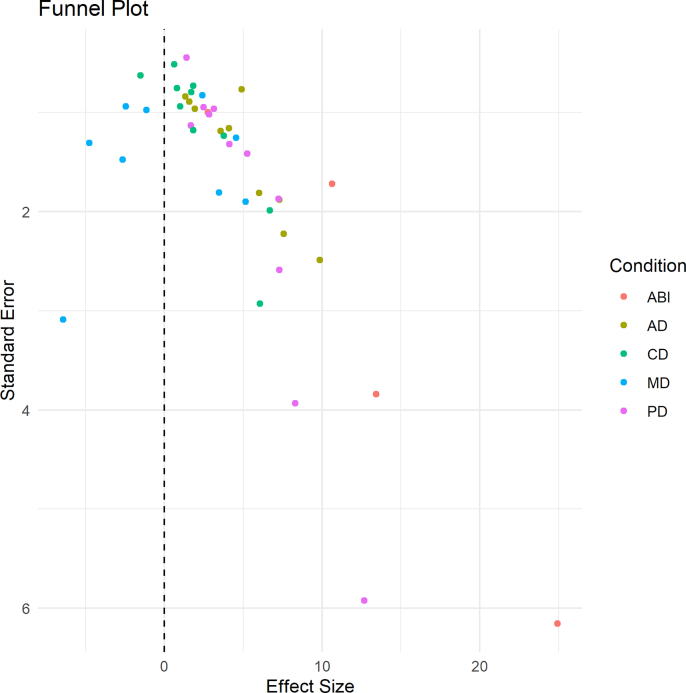


## Data Availability

Data will be made available on request.

## Glossary

AD: Alzheimer's disease
ARDS: acute respiratory distress syndrome
BDE47: 2,2′,4,4′-tetra-bromodiphenyl ether
BBB: blood-brain barrier
CI: confidence interval
CNS: central nervous system
CRS: chronic restraint stress
CSDS: chronic social defeat stress
CUMS: chronic unpredictable mild stress
EC: entorhinal cortex
EPM: elevated plus maze
FMT: faecal microbiota transplantation
FST: forced swim test
GF: germ-free
GFAP: glial fibrillary acidic protein
GM: gut microbiota
HFD: high-fat diet
Hip: hippocampus
ICH: intracerebral haemorrhage
ICV: intracerebroventricular
IF: Immunofluorescence
IHC: immunohistochemistry
IL-1B: Interleukin 1 beta
LDL: low-density lipoprotein
LPS: lipopolysaccharide
MCAO: middle cerebral artery occlusion
MGA: microbiota-gut-astrocyte
MPTP: 1-methyl-4-phenyl-1,2,3,6-tetrahydropyridine
MWM: Morris water maze
NOR: novel object recognition
OMVs: outer membrane vesicles
PD: Parkinson's disease
PFC: prefrontal cortex
PRISMA: Preferred Reporting Items for Systematic Reviews and Meta-Analyses
SCFAs: short-chain fatty acids
SN: substantia nigra
SPF: specific pathogen-free
SPT: sucrose preference test
T2DM: type 2 diabetes mellitus
TBI: traumatic brain injury
TCM: traditional Chinese medicine
TLR4: toll-like receptor 4
TMAO: trimethylamine N-oxide
TNF: Tumour necrosis factor
TST: tail suspension test
VTA: ventral tegmental area
