# Supplementary material for: Systematic review and meta-analysis of microbiota-gut-astrocyte axis perturbation in neurodegeneration, brain injury, and mood disorders
Source: Brain Behav Immun Health. 2025 May 12;46:101013. doi: 10.1016/j.bbih.2025.101013 (PMC12145769; doi:10.1016/j.bbih.2025.101013)
Supplement: Multimedia component 4 [file mmc4.docx]

| **Section and Topic** | **Item #** | **Checklist item** | **Location where item is reported** |
| --- | --- | --- | --- |
| **TITLE** | | |  |
| Title | 1 | Identify the report as a systematic review. | Yes pg. 1 |
| **ABSTRACT** | | |  |
| Abstract | 2a | Identify the report as a systematic review | Yes pg. 1 |
|  | 2b | Provide an explicit statement of the main objective(s) or question(s) the review addresses | Yes pg. 1 |
|  | 2c | Specify the inclusion and exclusion criteria for the review | Yes pg. 1 |
|  | 2d | Specify the information sources (such as databases, registers) used to identify studies and the date when each was last searched | Yes pg. 1 |
|  | 2e | Specify the methods used to assess risk of bias in the included studies | Yes pg. 1 |
|  | 2f | Specify the methods used to present and synthesise results | Yes pg. 1 |
|  | 2g | Give the total number of included studies and participants and summarise relevant characteristics of studies | Yes pg.1 |
|  | 2h | Present results for main outcomes, preferably indicating the number of included studies and participants for each. | Yes pg.1 |
|  | 2i | Provide a brief summary of the limitations of the evidence included in the review (such as study risk of bias, inconsistency, and imprecision) | Yes pg. 1 |
|  | 2j | Provide a general interpretation of the results and important implications | Yes pg.1-2 |
|  | 2k | Specify the primary source of funding for the review | NA |
|  | 2l | Provide the register name and registration number | NA |
| **INTRODUCTION** | | |  |
| Rationale | 3 | Provide an explicit statement of the objective(s) or question(s) the review addresses. | Yes p.4 |
| Objectives | 4 | Provide an explicit statement of the objective(s) or question(s) the review addresses. | Yes p.4 |
| **METHODS** | | |  |
| Eligibility criteria | 5 | Specify all databases, registers, websites, organisations, reference lists and other sources searched or consulted to identify studies. Specify the date when each source was last searched or consulted. | Yes p.4 |
| Information sources | 6 | Present the full search strategies for all databases, registers and websites, including any filters and limits used. | Yes p.4 |
| Search strategy | 7 | Specify the methods used to decide whether a study met the inclusion criteria of the review, including how many reviewers screened each record and each report retrieved, whether they worked independently, and if applicable, details of automation tools used in the process. | Yes p.5 |
| Selection process | 8 | Specify the methods used to collect data from reports, including how many reviewers collected data from each report, whether they worked independently, any processes for obtaining or confirming data from study investigators, and if applicable, details of automation tools used in the process. | Yes p.5 |
| Data collection process | 9 | List and define all outcomes for which data were sought. Specify whether all results that were compatible with each outcome domain in each study were sought (e.g. for all measures, time points, analyses), and if not, the methods used to decide which results to collect. | Yes pg. 6 |
| Data items | 10a | List and define all other variables for which data were sought (e.g. participant and intervention characteristics, funding sources). Describe any assumptions made about any missing or unclear information. | Yes pg. 6 |
|  | 10b | Specify the methods used to assess risk of bias in the included studies, including details of the tool(s) used, how many reviewers assessed each study and whether they worked independently, and if applicable, details of automation tools used in the process. | Yes pg. 5 |
| Study risk of bias assessment | 11 | Specify for each outcome the effect measure(s) (e.g. risk ratio, mean difference) used in the synthesis or presentation of results. | Yes pg. 6 |
| Effect measures | 12 | Describe the processes used to decide which studies were eligible for each synthesis (e.g. tabulating the study intervention characteristics and comparing against the planned groups for each synthesis (item #5)). | Yes pg. 4-5 |
| Synthesis methods | 13a | Describe any methods required to prepare the data for presentation or synthesis, such as handling of missing summary statistics, or data conversions. | Yes pg. 6 |
|  | 13b | Describe any methods used to tabulate or visually display results of individual studies and syntheses. | Yes pg. 6 |
|  | 13c | Describe any methods used to synthesize results and provide a rationale for the choice(s). | Yes pg. 6 |
|  | 13d | Describe any methods used to explore possible causes of heterogeneity among study results (e.g. subgroup analysis, meta-regression). | Yes pg. 6 |
|  | 13e | Describe any sensitivity analyses conducted to assess robustness of the synthesized results. | NA |
|  | 13f | Describe any methods used to assess risk of bias due to missing results in a synthesis (arising from reporting biases). | Table S1, Figure S1 |
| Reporting bias assessment | 14 | Describe any methods used to assess certainty (or confidence) in the body of evidence for an outcome. | Table S1, Figure S1 |
| Certainty assessment | 15 | Describe any methods used to assess certainty (or confidence) in the body of evidence for an outcome. | Table S1, Figure S1 |
| **RESULTS** | | |  |
| Study selection | 16a | Cite studies that might appear to meet the inclusion criteria, but which were excluded, and explain why they were excluded. | Yes pg. 7 |
|  | 16b | Cite each included study and present its characteristics. | Yes p.6-8 |
| Study characteristics | 17 | Present assessments of risk of bias for each included study. | Table S1 |
| Risk of bias in studies | 18 | For all outcomes, present, for each study: (a) summary statistics for each group (where appropriate) and (b) an effect estimate and its precision (e.g. confidence/credible interval), ideally using structured tables or plots. | Figures 2-4 |
| Results of individual studies | 19 | For each synthesis, briefly summarise the characteristics and risk of bias among contributing studies. | Yes p.6-8 |
| Results of syntheses | 20a | Present results of all statistical syntheses conducted. | Yes pg.10-14 |
|  | 20b | Present results of all investigations of possible causes of heterogeneity among study results. | Yes pg.8 |
|  | 20c | Present results of all sensitivity analyses conducted to assess the robustness of the synthesized results. | NA |
|  | 20d | Present assessments of risk of bias due to missing results (arising from reporting biases) for each synthesis assessed. | Table S1, Figure S1 |
| Reporting biases | 21 | Present assessments of certainty (or confidence) in the body of evidence for each outcome assessed. | Table S1, Figure S1 |
| Certainty of evidence | 22 | Present assessments of certainty (or confidence) in the body of evidence for each outcome assessed. | Table S1, Figure S1 |
| **DISCUSSION** | | |  |
| Discussion | 23a | Discuss any limitations of the evidence included in the review. | Yes pg. 16 |
|  | 23b | Discuss any limitations of the review processes used. | Yes pg. 16 |
|  | 23c | Discuss implications of the results for practice, policy, and future research. | Yes p.14-15 |
|  | 23d | Discuss implications of the results for practice, policy, and future research. | Yes p.14-15 |
| **OTHER INFORMATION** | | |  |
| Registration and protocol | 24a | Provide registration information for the review, including register name and registration number, or state that the review was not registered. | NA |
|  | 24b | Indicate where the review protocol can be accessed, or state that a protocol was not prepared. | NA |
|  | 24c | Describe and explain any amendments to information provided at registration or in the protocol. | NA |
| Support | 25 | Describe sources of financial or non-financial support for the review, and the role of the funders or sponsors in the review. | NA |
| Competing interests | 26 | Declare any competing interests of review authors. | Yes pg. 2 |
| Availability of data, code and other materials | 27 | Report which of the following are publicly available and where they can be found: template data collection forms; data extracted from included studies; data used for all analyses; analytic code; any other materials used in the review. | All data included in ms and supporting files |

*From:*  Page MJ, McKenzie JE, Bossuyt PM, Boutron I, Hoffmann TC, Mulrow CD, et al. The PRISMA 2020 statement: an updated guideline for reporting systematic reviews. BMJ 2021;372:n71. doi: 10.1136/bmj.n71
